# Reversible congenital hypogonadotropic hypogonadism: keys for clinical management

**DOI:** 10.20945/2359-4292-2026-0063

**Published:** 2026-05-01

**Authors:** Chrysoula Archaki, Maria Stamou, Andrew A. Dwyer

**Affiliations:** 1 Aristotle University of Thessaloniki, School of Medicine, Thessaloniki, Greece; 2 Massachusetts General Hospital, Reproductive Endocrine Unit and P50 Massachusetts General Hospital - Harvard Center for Reproductive Medicine, Boston, Massachusetts, USA; 3 Boston College William F. Connell School of Nursing and P50 Massachusetts General Hospital - Harvard Center for Reproductive Medicine, Chestnut Hill, Massachusetts, USA

**Keywords:** Kallmann syndrome, male infertility, male reproduction, testosterone replacement

## Abstract

Congenitalhypogonadotropic hypogonadism (CHH) is characterized by absent/incomplete puberty and a treatable form of male infertility resulting from deficient gonadotropin-releasing hormone (GnRH) secretion/action. Traditionally, CHH has been considered a permanent, lifelong condition. However, evidence indicates a subset men undergo reversal and recover reproductive axis function. We conducted a structured literature search (Medline, PubMed) using keywords to retrieve articles on CHH reversal (1975-2025). We synthesize the literature to provide a high-level overview of CHH and the reversal phenomenon in males. Particular focus is given to clinical aspects of CHH and reversal using a case vignette to highlight keys to management. Approximately 10-15% of males with CHH undergo reversal with sustained normalized testosterone levels and spermatogenesis off treatment. A key sign of reversal is testicular growth while on testosterone replacement therapy. Those men with some degree of spontaneous puberty (i.e., larger testicular volume), detectable follicle stimulating hormone levels , and/or pathogenic variants in *GNRHR* are more likely to undergo reversal. In contrast, severe GnRH deficiency, pathogenic *ANOS1* and/or two or more combined pathogenic variants (oligogenicity) in CHH genes are less likely to recover reproductive axis function. Reversal is not lasting in all cases. The reversal phenomenon challenges the classical dogma that CHH is permanent and lifelong. Reversal cases highlight the plasticity of the neuroendocrine control of reproduction. Clinicians can tailor the approach to men with CHH using reversal predictors to guide supervise washout to identify reversal as well as long-term monitoring to assess potential relapse.

**Clinical Vignette:** A 19-year-old male presented for endocrine consultation and evaluation of delayed puberty. His history was unremarkable but for anosmia (noted at age 10 yrs.) and failure to spontaneously initiate puberty. At presentation, he was normal weight (BMI 23.1 kg/M^2)^ and his exam was notable for lack of virilization, Tanner II pubic hair, and small testes (5 mL bilaterally). Biochemical evaluation showed normal TSH, prolactin, and IGF1 levels - yet low serum gonadotropins (LH = 1.2 IU/L [ref: 1.6-8.0], FSH = 1.8 [ref: 1.5-9.3]) in the setting of frankly hypogonadal serum testosterone (T) (33 ng/dL [ref: 300-1,000]). The clinical and biochemical findings were consistent with Kallmann syndrome (i.e., congenital hypogonadotropic hypogonadism [CHH] with anosmia) and he initiated T enanthate injections. The dose was increased progressively, and he achieved normal serum T levels on a full adult dose (200 mg IM every two weeks). At his 6-month clinical follow-up visit, his testicular volume was noted to have increased to 9 mL bilaterally. He was instructed to stop treatment and serial monitoring demonstrated sustained, normal serum gonadotropin levels (LH: 5.5 IU/L, FSH: 3.6 IU/L) and eugonadal T (434 ng/dL) - consistent CHH reversal. Three months after discontinuing treatment, his testes had grown to 12 mL bilaterally and seminal fluid analysis revealed sperm in the ejaculate (0.4 X10^6^/mL [ref: >15.0]).

## INTRODUCTION

Puberty is one of the most striking periods of human life. In males, the most important physiological changes of puberty include accelerated linear growth, changes in body composition, development of secondary sex characteristics, and testicular enlargement. A range of hereditary and environmental factors influence the timing of pubertal onset ^(1)^. Delayed puberty in males is defined as lack of testicular development (testicular volume <4 mL) by the age of 14 years. In self-limited delayed puberty (SLDP), puberty begins later than peers yet occurs spontaneously and progresses to completion without needing intervention. In contrast, individuals with congenital hypogonadotropic hypogonadism (CHH) do not complete puberty on their own ^(2)^. Currently, there is no ‘gold standard’ test to differentiate SLDP from CHH, so it remains a diagnosis of exclusion, and CHH is often diagnosed late ^(3)^ - often contributing to psychosocial morbidity and diminished health-related quality of life ^(4,5)^. Clinically, CHH is characterized by absent or incomplete pubertal development and infertility. It is caused by isolated deficiency in secretion(or action) of gonadotropin releasing hormone (GnRH) - a key regulator of the hypothalamic-pituitary-gonadal (HPG) axis that governs reproduction ^(2)^. Biochemically, CHH is defined by low (or inappropriately normal) serum gonadotropin levels in the setting of low serum sex steroids and otherwise normal pituitary imaging and function ^(6)^. Notably, CHH is rare (1:48,000) and there is a 3:1 ratio of affected males to females ^(7)^. More than 60 genes have been identified to underlie CHH yet the known genes only account for about half of cases ^(8)^. Sex steroids are effective for inducing secondary sexual characteristics and fertility can be induced in about 75-80% of cases ^(9)^ using either gonadotropin therapy or pulsatile GnRH ^(10)^. Despite the notion that congenital disorders are lifelong, approximately 10%-15% of males with CHH experience a reversal and recover reproductive axis function with normalized serum testosterone and spermatogenesis ^(11,12)^. The reversal phenomenon highlights the plasticity of the hypothalamic neurons and the possible role of other neuronal populations in regulating the reproductive axis ^(12)^. Reversal has been observed in individuals harboring rare pathogenic variants in CHH genes suggesting the contribution of epigenetic factors in CHH reversal ^(13)^. This review provides an overview of the approach to managing the male patient with CHH with particular emphasis on the reversal phenomenon.

## MATERIALS AND METHODS

We conducted a structured, systematic literature search using Medline and PubMed to identify relevant articles on CHH reversal published in English between 1975-2026. Keywords (i.e., Medical Subject Headings - MeSH terms) included “hypogonadism” or “Kallmann” and “reversal” or “recovery”. Retrieved articles underwent title and abstract screening. Full-text review was used to exclude articles that did not include CHH cases, reports on adult-onset hypogonadism (i.e., complete spontaneous puberty), and functional hypogonadism (i.e., obesity or exercise-related). Peer-reviewed case reports, original research articles, and review articles documenting reversal of CHH were included for synthesis in this review.

### Reproductive axis

**Figure 1:**
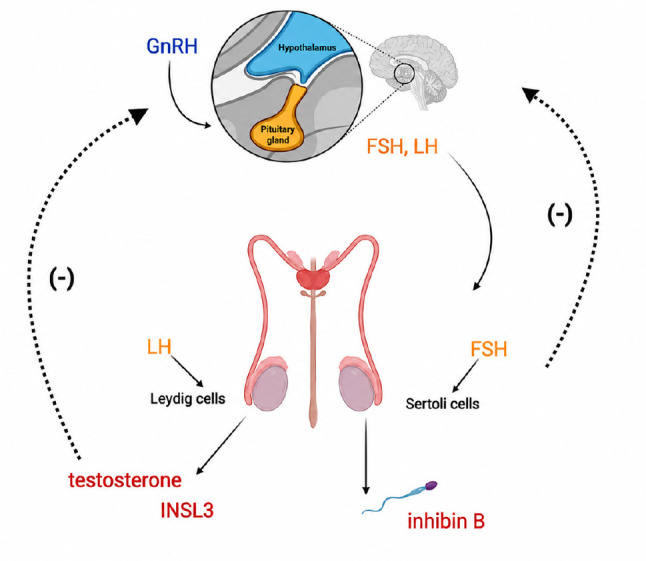
Schematic depicting the hypothalamic-pituitary-gonadal axis.

Specialized GnRH-secreting neurons regulate the hypothalamic-pituitary-gonadal (HPG) axis and human reproduction. Around 14-weeks of gestation, GnRH neurons migrate from their initial location in the olfactory epithelium (embryonic olfactory placode) crossing the cribriform plate and past the developing olfactory bulb to the hypothalamus. There are only approximately 1,200-1,500 GnRH neurons ^(2)^ and the pulsatile secretion of GnRH triggers pituitary gonadotrophs to release luteinizing hormone (LH) and follicle-stimulating hormone (FSH). Gonadotropins (LH and FSH) stimulate the gonads to produce sex steroids and gametes. In males, LH acts on testicular Leydig cells stimulating testosterone (T) production while FSH acts on testicular Sertoli cells stimulating inhibin B secretion and spermatogenesis. Sex steroids and inhibin B regulate the HPG axis through negative feedback (**Figure 1**) ^(2)^.

Created in BioRender. Archaki, C. (2025) https://BioRender.com/undefined

Pulsatile GnRH secretion stimulates the release of LH and FSH from pituitary gonadotrophs. In males, LH acts on testicular Leydig cells spurring testosterone (T) production while FSH supports spermatogenesis and stimulates testicular Sertoli cells to produce inhibin B. Both T and inhibin B exert negative feedback on the reproductive axis. GnRH: gonadotropin-releasing hormone; FSH: follicle-stimulating hormone; LH: luteinizing hormone; INSL3: insulin-like peptide 3.

There are three critical waves of HPG activation during development. The first activation occurs *in utero*. In males, maternal human chorionic gonadotropin (hCG) and fetal HPG axis activation stimulate Leydig cells to produce T and insulin-like peptide 3, resulting in normal penile development and the final phase of testicular descent, respectively. The second wave, or so-called minipuberty, occurs during the first 4-6 months of neonatal life. This brief window of HPG axis activation drives Sertoli and germ cell proliferation and is a key early-life determinant of future reproductive capacity ^(14)^. The third wave of HPG axis activation occurs at the onset of puberty, which is characterized by the development of secondary sexual characteristics and full reproductive capacity.

### Presentation and diagnosis of CHH

The clinical presentation of CHH is heterogeneous ranging from isolated GnRH deficiency to syndromic forms with variable constellations of non-reproductive phenotypes (i.e., sensory, dental, muculoskeletal, neurologic) (**Table 1**) ^(2,15)^. Approximately half of cases exhibit non-reproductive phenotypes, the most common being lack of sense of smell (CHH + anosmia = Kallmann syndrome). Moreover, the degree of GnRH deficiency can be variable from absent minipuberty (e.g., cryptorchidism with/without micropenis) with complete absent of spontaneous puberty (i.e., testicular volume [TV] <4 mL) to partial spontaneous puberty (TV ≥4 mL) and the Pasqualini syndrome characterized by CHH with near-complete puberty development (TV: 8-10 mL) ^(16)^. Ultimately, CHH is a clinical diagnosis of exclusion yet the variable clinical presentation as well as lack of a ‘gold standard’ test to differentiate SLDP from CHH contribute to late diagnosis (i.e., at 18 yrs or later) ^(2,4)^.

**Table 1:** CHH-associated phenotypes from the literature

Reproductive	Non-Reproductive (syndromic)		
Cryptorchidism Micropenis Absent minipuberty Absent puberty Partial puberty Reversal of CHH	**Sensory & Neurologic** Anosmia/hyposmia Hearing loss Ear anomalies Coloboma Ano/micropthalmia Synkinesia Cerebellar ataxia	**Skeletal & Dental** Osteopenia/-porosis Scoliosis Syndactyly Clinodactyly camptodactyly Split hand/foot Joint hyperlaxity Pectus excavatum Eunuchoidal proportions Missing/extra teeth	**Developmental & Other** Cleft lip/palate Bifid uvula Choanal atresia Renal agenesis Pituitary defects Arrhinia Ichthyosis Achromic hair patches Congenital heart defects Developmental delay Intellectual disability

Dynamic stimulation tests, such as GnRH stimulation and FSH-stimulated inhibin B, among others, have been proposed to differentiate CHH from SLDP, yet these tests lack sufficient specificity ^(17)^. Recent research has identified kisspeptin stimulation as a promising test for differentiating SLDP and CHH ^(18)^. Kisspeptin is a neuropeptide “upstream” of the hypothalamus that triggers the release of GnRH and activates the HPG axis in the onset of puberty ^(2)^. While initial study results are promising, further work is needed to definitively prove effectiveness as a clinical diagnostic test.

Notably, several clinical ‘red flags’ (cryptorchidism, micropenis, anosmia) can help speed diagnosis leading to timely treatment ^(19)^. The vast majority of CHH cases are sporadic with only about 30% showing familial inheritance ^(2,8)^. The genetics of CHH is complex with autosomal dominant, autosomal recessive, X-linked, digenic (i.e., two rare variants), and oligogenic forms occurring in roughly 15% of cases ^(20)^. To date, more than 60 genes have been identified to underlie CHH, accounting for approximately half of cases ^(8)^. As such, panel testing can inform molecular diagnosis of CHH and speed diagnosis in some cases, yet it is not universally informative ^(21)^. It is worthwhile to note that late diagnosis (“diagnostic odyssey”) is associated with psychosocial morbidity (e.g., anxiety, depression) ^(4)^, psychosexual sequelae ^(5)^ and diminished health-related quality of life ^(22)^. Evidence supports that earlier diagnosis enables timely initiation of treatment and helps alleviate some of the psychosocial impact and emotional distress of CHH ^(23)^.

### Treatment of CHH

Unlike many rare diseases, effective treatments are available for CHH. Exogenous testosterone (T) treatment (i.e., transdermal gels or long-acting injectable esters) is effective for inducing secondary sexual characteristics ^(24)^. Treatment is initiated with low dose (e.g., 50 mg IM monthly) then progressively titrated to full adult dosage with trough T levels at the lower end of the normal reference range. However, a more rapid dose escalation is typically used for males who present with CHH well into adulthood ^(24)^. Adherence to treatment should be assessed at every visit ^(6)^ and men will typically need to remain on lifelong T treatment using either a transdermal preparation, an injectable T ester (e.g., enanthate, cypionate, undecanoate), or implantable T pellets. While T treatment induces virilization and can normalize sexual function, it will not induce testicular development or fertility. Importantly, increased TV on T treatment indicates activation of the HPG axis and may suggest reversal of CHH.

Unlike many forms of male infertility, CHH is treatable and approximately 75%- 80% of males with CHH can develop sperm in the ejaculate using either pulsatile GnRH (via microinfusion pump) or gonadotropin therapy (human chorionic gonadotropin [hCG], FSH) ^(9)^. Pulsatile GnRH is typically limited to specialized research centers while gonadotropins are much more widely used. There are several options for inducing fertility in males with CHH, including concurrent administration of hCG with FSH or sequential gonadotropin therapy (i.e., unopposed FSH followed by hCG + FSH) ^(10)^. Monotherapy with hCG alone is not the preferred approach given suboptimal outcomes ^(25)^. Many men with CHH are able to achieve fertility within a year ^(26)^. Research has identified history of cryptorchidism, complete absence of spontaneous pubertal development (TV <4 mL), and low serum inhibin B (<60 pg/mL) as negative predictors of outcome to fertility-inducing treatment ^(27,28)^. In such cases, sequential gonadotropin therapy, which aims to recreate the hormonal milieu of minipuberty, appears to provide the best chances for developing fertility ^(10,29)^. Although most men with CHH will not achieve WHO-defined normal sperm counts (>20x10^6^/mL) ^(30)^, low sperm counts do not preclude fertility or conception ^(31)^. As previously noted, T treatment will not induce spermatogenesis, so fertility while on T should warrant evaluation of CHH reversal ^(12)^.

### CHH reversal

**Figure 2:**
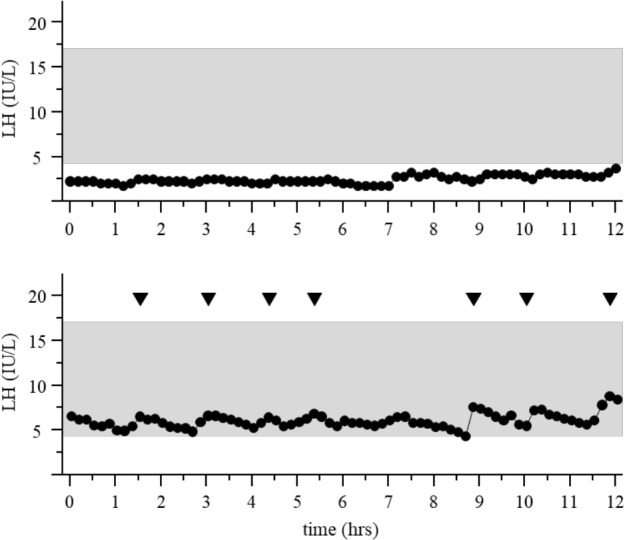
LH pulse study before and after reversal.

Traditionally, CHH has been considered a congenital, permanent condition requiring lifelong treatment. A 1975 case report raised the first notion that CHH may be reversible ^(32)^. Subsequently, additional case reports over the next decades documented men with confirmed CHH who recovered their HPG axis function with normalized serum T levels (**Supplemental Table 1**). In 2007, the first prospective study of the CHH reversal phenomenon identified five reversal cases among a cohort of 50 CHH men, suggesting a reversal rate of 10% ^(13)^. Reversal cases are heterogeneous. Some men who undergo CHH reversal only have isolated GnRH deficiency while others exhibit a constellation of non-reproductive phenotypes ^(11,12)^. Some men exhibit signs of absent minipuberty and a complete absence of spontaneous pubertal puberty (TV < 4 mL) ^(33)^ while others exhibit the relatively mild GnRH deficiency with partial spontaneous puberty (i.e., Pasqualini syndrome) ^(16)^. While clinical presentation varies across reversal cases, all share a similar pattern - HPG axis activation (**Figure 2**) following a period of normalized serum sex steroid levels from either exogenous T, gonadotropins, or pulsatile GnRH therapy ^(11,12)^.

Panels depict serum LH levels during overnight (17:30-05:30) 12-hour frequent blood sampling studies (Q 10 min. x 12 hrs.). Upper panel: At diagnosis, the 19-year-old male exhibited apulsatile LH secretion and frankly hypogonadal testosterone (33 ng/dL). Testosterone therapy was initiated and continued for 18 months. The patient’s testicular volume had increased from 4 mL to 6 mL on testosterone. Treatment was discontinued and he underwent a repeat frequent sampling study (lower panel) revealing LH pulses (inverted triangles) in the setting of normal serum testosterone levels (334 ng/dL). Gray shaded region depicts the mean LH ± 2 S.D.s of healthy volunteers ^(13)^.

A 2024 study of 87 reversal cases across six international referral centers identified two distinct classes of reversals and several predictors ^(34)^. Men were more likely to undergo reversal if they exhibited milder GnRH deficiency (i.e., Pasqualini syndrome) with no micropenis, some degree of spontaneous testicular development (TV ≥8 mL), and detectable FSH (i.e., ~4.0 IU/L). Prior work conducting detailed neuroendocrine profiling (i.e., 12-hr LH pulsatility studies) found a LH threshold of 2.1 IU/L being a fairly reliable proxy for pulsatile LH secretion ^(15)^. That data was in line with the results of a 2015 Chinese study that identified 18 reversals (5.1%) in a cohort of 354 males with CHH study ^(35)^. Authors noted that larger testicular size and higher LH levels (both basal and tryptorelin-stimulated) were associated with reversal ^(35)^. However, the 2024 study of the largest reversal cohort (n=87) to date found no link between LH and reversal ^(34)^.

### Genetics of reversal

It is notable that reversal cases have been documented in individuals harboring pathogenic rare variants in CHH genes ^(11,12)^ (**Supplemental Table 1**). To date, rare variants in more than 30 CHH genes have been found in cases with CHH reversal (**Table 2**). Genetic factors that can help us determine the likelihood of CHH reversal include: (i) variants in the autosomal recessive GnRH receptor gene (*GNRHR*). Recent data found that men who harbor pathogenic *GNRHR* variants are more likely to undergo reversal ^(34)^; (ii) variants in the X-linked gene, *ANOS1*. Among the largest reversal cohort studied to date (n=87), no pathogenic variants in *ANOS1* were detected. Notably, *ANOS1* variants cause a particularly severe form of GnRH deficiency ^(15,36)^ that is rather unresponsive to fertility-inducing treatment ^(10,15)^. Curiously, a 2007 case report described a reversal in a male harboring an *ANOS1* - then termed *KAL1* ^(37)^. Several factors raise questions about the validity of the *ANOS1* reversal case report. First, sequencing technology has advanced significantly and has since reduced the risk of pseudogene findings that were once more likely in *ANOS1* sequencing. Second, the patient’s T level at diagnosis was 212 ng/dL - which is uncharacteristically higher than expected in light of the severe *ANOS1*-related GnRH deficiency. Third, no other cases of *ANOS1*-associated reversal have been documented (Supplemental Table 1). Thus, patients harboring *ANOS1* variants are unlikely to undergo reversal; (iii) The genetic load observed in patients screened. Men with pathogenic variants in more than one gene (i.e., oligogenicity) are less likely to undergo reversal ^(34)^.

**Table 2:** CHH Genes identified in reversal grouped according to gene function

GnRH neuron migration	GnRH secretion	CHH & other overlapping syndromes
*ANOS1* (XLR) *CCDC141* (AR) *DCC* (AD) *FEZF1* (AR) *FGF8* (AD) *FGFR1* (AD) *HS6ST1* (AD) *IL17RD* (AR) *NSMF* (AR) *PLXNA1* (AD) *PROK2* (AR) *PROKR2* (AR) *SEMA3A* (AD) *SEMA7A* (AD)	*AMH* (AR) *DMXL2* (AR) *GNRH1* (AR) *IGSF10* (AD) *KISS1* (AR) *KISS1R* (AR) *LEPR* (AR) *TAC3* (AR) *TAC3R* (AR)	CHARGE syndrome *• CHD7* (AD) Combined pituitary hormone deficiency *• SOX2* (AD) *• WDR11* (AD) Gordon Holmes Syndrome *• OTUD4* (AR) *• PNPLA6* (AR) *• RNF216* (AR) Pallister-Hall Syndrome *• GLI3* (AD) Waardenburg Syndrome (Type 2) *• SOX10* (AD) Warburg Micro Syndrome *• RAB3GAP1* (AR) *• RAB3GAP2* (AR)
	GnRH action	
	*GNRHR* (AR)	

AD: autosomal dominant; AR: autosomal recessive; XLR: X-linked recessive.

Of note, when genetic testing is unavailable or cost-prohibitive, several key factors can increase our suspicion for an *ANOS1* variant, and thus, decrease the likelihood of CHH reversal ^(34)^. First, *ANOS1* invariably causes Kallmann syndrome, so complete anosmia is an essential hallmark of *ANOS1* variants. Individuals with *ANOS1* variants are more likely to exhibit mirror movements (i.e., synkinesia) and have renal agenesis ^(38)^. Further, as *ANOS1* follows an X-linked recessive inheritance. Thus, careful family history and pedigree analysis can provide critical clues. Last, CHH caused by *ANOS1* variants is typically a severe form of GnRH deficiency and is associated with cryptorchidism (with/without micropenis) and complete absence of spontaneous puberty (TV <4 mL) ^(15)^.

### Proposed mechanisms of reversal

The mechanism(s) underlying CHH reversal remain unclear. The 2007 prospective study was the first reversal study to report with a pathogenic variant in a CHH gene ^(13)^. Authors hypothesized that genetic defects could affect GnRH neuron maturation, and androgens could upregulate genes involved in synchronized GnRH secretion. Additional support for the proposed mechanism came in a subsequent report of two men who were able to sustain HPG axis function (i.e., CHH reversal) reversal with intermittent exogenous androgen exposure ^(39)^. To date, a number of factors have been proposed, including epigenetic factors, non-coding regions of the genome, and other neuronal populations in the hypothalamus (astrocytes, tanycytes) that interact with GnRH neurons via KNDy neurons (kisspeptin, neurokinin B, and dynorphin) ^(40)^. Kisspeptin is a potent stimulator of GnRH secretion and it is well known that sex steroids exert feedback effects regulating the GnRH pulse pattern ^(41,42)^. Thus, CHH reversal appears to be related to the plasticity of the neuronal network involved in GnRH secretion.

### Relapse of reversal

It is important to underscore that the recovery of HPG axis function may not be lasting, and some patients revert to a hypogonadal state. According to a large retrospective study of 308 patients with CHH, 39 men underwent reversal and 5/39 (13%) of them experienced a subsequent relapse to a hypogonadal state ^(43)^. Common characteristics among those who relapsed to a hypogonadal state included a significant stressor - either emotional, psychiatric, or metabolic. Such stressors can disrupt pubertal timing and development ^(44)^. Indeed, anxiety/depression and changes in energy balance can impair the reproductive axis with suppressed T levels and decreased LH pulse frequency/amplitude - akin to hypothalamic amenorrhea in females ^(45)^. Thus, ongoing surveillance and hormonal monitoring is warranted following reversal ^(2,6,11,12)^. If spermatogenesis is achieved during a window of CHH reversal, sperm banking should be offered to mitigate the loss of fertility in the event of relapse. The dynamic nature of the HPG axis is highlighted by individuals who undergo a ‘second’ reversal (i.e., T = 257 ng/dL) after relapse and recover reproductive function a second time ^(43)^.

### Return to case

Positive predictors for the patient’s reversal include his partial puberty (with baseline TV of 5 mL bilaterally), detectable FSH and lack of severe GnRH deficiency during mini puberty (no cryptorchidism or micropenis). Following reversal, the patient returned to clinic every six months for ongoing surveillance. At the 18-month visit he complained of fatigue and decreased frequency and strength of his morning erections. Blood work showed hypogonadal T (205 ng/dL). Treatment options were discussed with the patient (i.e., T replacement vs. gonadotropin injections) and the patient opted to cryopreserve sperm, his current sperm count was now 1.7X10^6^/mL, then initiate T replacement with ongoing monitoring.

## CONCLUSION

In summary, CHH is clinically and genetically heterogenous and there are a number of key facets of comprehensive disease management (**Inset Box 1**). While CHH was traditionally considered lifelong, we know that approximately 10-15% of men undergo reversal, with recovery of HPG axis function following a period of normalized T levels ^(12)^. Increase in testicular size, in conjunction with the normalization of circulating levels of sex steroids while on T replacement is a reliable hallmark ^(11,12)^. Given the possibility of reversal, clinicians can consider key factors favoring reversal to target supervised treatment washouts every 1-2 years. Key signs that increase the likelihood of reversal include: history of partial puberty (TV >4 mL), detectable gonadotropins (FSH ~ 4 IU/L), and/or variant in *GNRHR.* On the other hand, signs suggestive of severe GnRH deficiency and absent minipuberty (i.e., cryptorchidism with/without micropenis) as well as pathogenic variants in *ANOS1* and/or variants in two or more CHH genes (i.e., oligogenicity) decrease the likelihood of reversal. Notably, reversal is not always permanent, so longitudinal monitoring is warranted. In conclusion, the reversal phenomenon challenges the dogma that congenital genetic conditions are lifelong and reversible CHH raises many questions regarding neuronal plasticity and epigenetic influences on the neuroendocrine control of reproduction.

**Inset Box 1:**
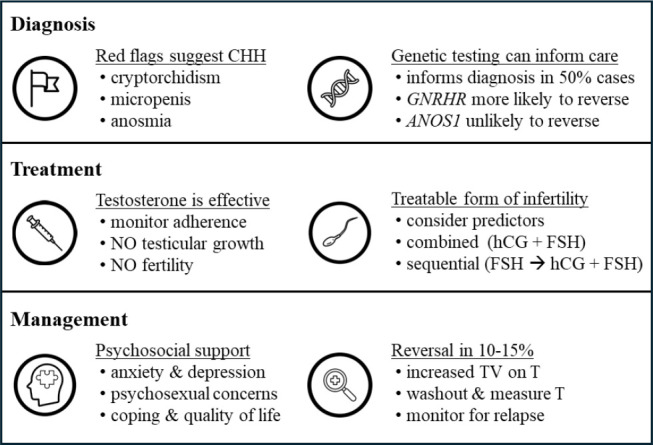
Key points for diagnosis, treatment, and management of CHH

## Figures and Tables

**Supplemental Table 1:** Reports of CHH reversal (1975-2025)

Year (ref.)	Article type (# cases)	Notes (genes reported)
1975 ^(1)^	case report (n=1)	first case report, anosmic (KS), TRT only, increased TV, spermatogenesis
1980 ^(2)^	case series (n=2)	both anosmic with Pasqualini syndrome, conceived with proven paternity
1983 ^(3)^	case report (n=1)	anosmic (KS), TRT only (21 mos.), conceived with proven paternity
1986 ^(4)^	case report (n=1)	anosmic (KS), TRT only, increased TV, normal T sustained off treatment s/p 7 yrs.
1989 ^(5)^	cohort (n=4)	LH pulses in 4/10 CHH off long-term pulsatile GnRH therapy
1994 ^(6)^	case report (n=1)	anosmic (KS), hCG then TRT, spermatogenesis & normal T sustained off treatment, conception
1995 ^(7)^	case report (n=1)	anosmic with Pasqualini syndrome, TRT only, increased TV & spermatogenesis, conceived
1996 ^(8)^	cohort (n=1)	anosmic (KS), TRT only, LH pulses, normal T sustained off treatment
1999 ^(9)^	case series (n=5)	all anosmic (KS), increased TV on TRT, spermatogenesis documented in 4/5
2007 ^(10)^	cohort (n=15)	prospective study, 5/50 with normalized T off long-term treatment (*GNRHR, FGFR1*)
2007 ^(11)^	case report (n=1)	all anosmic (KS), increased TV on TRT, spermatogenesis, conceived (*ANOS1*)
2008 ^(12)^	case report (n=1)	anosmic (KS), sustained normalized T & spermatogenesis off treatment (*PROKR2*)
2010 ^(13)^	cohort (n=6)	reversals reported as part of a gene discovery project; reversal = LH pulsatility, increased TV and T, or spermatogenesis (*TAC3, TACR3*)
2011 ^(14)^	cohort (n=1)	reversals reported as part of a gene discovery project; anosmic (KS), normal T sustained off treatment for several years (*HS6ST1*)
2012 ^(15)^	cohort (n=6)	cohort of reversal cases (anosmic: n=2, normosmic: n=4) increased TV, normalized T & spermatogenesis off treatment (*CHD7, FGFR1, GNRHR*)
2013 ^(16)^	case report (n=1)	hCG then TRT, spermatogenesis & normal T sustained off treatment (*GNRHR*)
2013 ^(17)^	case series (n=6)	TRT only, increased TV, normal T sustained off treatment
2013 ^(18)^	cohort (n=2)	reversals reported as part of a FGF synexpression group gene discovery project(*IL17RD, SPRY4*)
2014 ^(19)^	cohort (n=18)	cohort of reversal cases (anosmic: n=5, normosmic: n=13) mix of hCG & TRT treatment, increased TV, normal T sustained off treatment
2014 ^(20)^	cohort (n=44)	cohort of reversal cases, reversal & relapse, first report o (*FGFR1, GNRHR, PROKR2, TACR3, TAC3,HS6ST1, NELF*)
2014 ^(21)^	case series (n=2)	both anosmic (KS), TRT only, increased TV, required intermittent T “booster’ to trigger reversal after relapse
2014 ^(22)^	cohort (n=1)	reversals reported as part of a gene discovery project; normal T sustained off treatment (*GNRHR*)
2016 ^(23)^	case report (n=1)	anosmic (KS), TRT only, increased TV, normal T & spermatogenesis sustained off treatment (*SOX10*)
2017 ^(24)^	case report (n=1)	Pasqualini syndrome, TRT only, increased TV, normal T sustained off treatment
2017 ^(25)^	case report (n=1)	anosmic (KS), TRT only, increased TV, normal T sustained off treatment (*PROK2*)
2019 ^(26)^	cohort (n=6)	reversals reported as part of a gene discovery project (anosmic: n=3, normosmic: n=3) increased TV, normal T sustained off treatment, one relapse (*GNRHR, IGSF10*)
2020 ^(27)^	cohort (n=1)	reversals reported as part of a gene discovery project; normosmic female (*CCDC141*)
2022 ^(28)^	cohort (n=13)	reversal cases recorded in a large cohort of patients (anosmic: n=6, normosmic: n=7) increased TV, spermatogenesis and/or fertility, normal T sustained off treatment (*CHD7, IL17RD, PROKR2*)
2022 ^(29)^	case report (n=1)	increased TV, normal T sustained off treatment (*GNRHR*)
2022 ^(30)^	case report (n=1)	increased TV, spermatogenesis, normal T off treatment, multiple relapses
2022 ^(31)^	cohort (n=16)	reversal cases recorded in a cohort of patients with Pasqualini syndrome (n=38); increased TV, spermatogenesis and/or fertility, normal T off treatment (*FGFR1, GNRHR*)
2024 ^(32)^	cohort (n=87)	cohort of reversal cases (n=87), multicenter study, predictors of reversal (34 genes reported)
2025 ^(33)^	case report (n=2)	single consanguineous family, TRT only, increased TV, normal T sustained off treatment (*KISS1*)

GnRH: gonadotropin-releasing hormone; hCG: human chorionic gonadotropin; KS: Kallmann syndrome; T: testosterone; TRT: testosterone replacement therapy; TV: testicular volume.

## Data Availability

datasets related to this article will be available upon request to the corresponding author.
